# A Degradomic Landscape of Proteolytic Remodeling in Melanoma Lung Metastasis

**DOI:** 10.1021/acs.jproteome.5c01205

**Published:** 2026-02-25

**Authors:** Camila Eduarda Alves Martins Nogueira, Fabiana Olímpio, Murilo Salardani, Uilla Barcick, Luis Roberto Fonseca, Flávio Aimbire, Letícia Dias Lima Jedlicka, Flávio V. Loures, Luciane Portas Capelo, Valdeci Lima, Taysa Monteiro de Oliveira, Bianca C. S. C. Barros, Solange M. T. Serrano, André Zelanis

**Affiliations:** † Functional Proteomics Laboratory, Federal University of São Paulo − UNIFESP, São José dos Campos, São Paulo 12231-280, Brazil; ‡ Department of Medicine, Postgraduate Program in Translational Medicine, Federal University of São Paulo (UNIFESP), Rua Pedro De Toledo 720−2° Andar, Vila Clementino, São Paulo, SP 04039-002, Brazil; § Department of Science and Technology, Lab. Immunopharmacology, Federal University of São Paulo (UNIFESP), Rua Talim, 330, Vila Nair, São José dos Campos, SP 12231-280, Brazil; ∥ Instituto de Estudos em Saúde e Biológicas, Universidade Federal do Sul e Sudeste do Pará- Unifesspa, 68507-590 Marabá, Pará, Brazil; ⊥ Applied Immunology Laboratory, Institute of Science and Technology, Federal University of São Paulo, Rua Talim, 330, Vila Nair, São José dos Campos, SP 12231-280, Brazil; # Laboratory of Bone Development and Bioengineering, Federal University of São Paulo − UNIFESP, São José dos Campos, São Paulo 12231-280, Brazil; 7 Laboratory of Applied Toxinology, Center of Toxins, Immune-Response, and Cell Signaling (CeTICS), 196591 Butantan Institute, São Paulo 05503-900, Brazil; 8 Laboratory of Cellular Immunology and Biochemistry of Fungi and Protozoa, Postgraduate Program in Pharmaceutical Sciences, Federal University of Sao Paulo (UNIFESP), Diadema, São Paulo 09913-030, Brazil

**Keywords:** melanoma, protease, proteolytic signaling, biomarkers, degradomics

## Abstract

Melanoma metastasis involves extensive remodeling of the tumor microenvironment, yet the proteolytic processes underlying pulmonary colonization remain poorly defined. Using the B16F10 intravenous melanoma model in C57BL/6J mice, we performed an integrative degradomic analysis of metastatic lung tissue, plasma, and secretome of early passaged primary cultures derived from metastatic foci. Semispecific database searches identified nearly 8,000 semitryptic peptides across compartments, with the secretome exhibiting the highest burden of cleavage events, indicating an intensely proteolytic microenvironment. Cleavage profiles were compartment-specific, dominated by actin in metastatic lung tissue, α2-macroglobulin in plasma, and SPARC in the secretome. Cleavage-site motif analysis revealed conserved His/Ser (P1/P1′) preferences in tissue and plasma, whereas the secretome showed a distinct Leu/Ser pattern. Discriminant-feature analysis uncovered unique proteolytic signatures for each compartment, and peptide-level mapping implicated at least 11 proteases—including MMP2, cathepsin D, and cathepsin E—as major contributors to metastatic niche remodeling. Functional enrichment demonstrated coordinated impacts on extracellular matrix organization, inflammation, and metabolic adaptation. This work provides a multicompartment degradomic resource that captures proteolytic remodeling in melanoma lung metastasis and establishes a foundation for future functional and translational studies.

## Graphical abstract

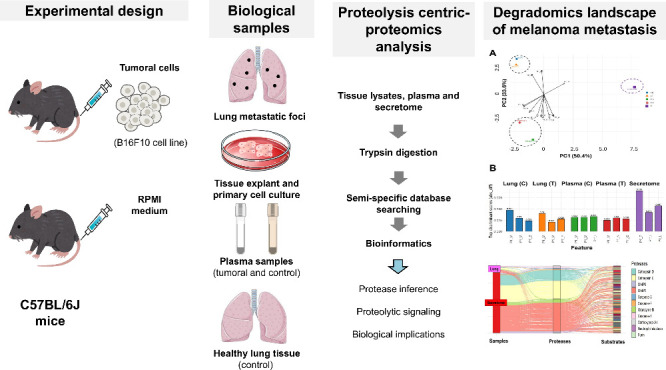

## Introduction

Cutaneous melanoma is an aggressive neoplasm arising from the uncontrolled proliferation of melanocytes and is associated with high mortality in advanced stages.^1^ Despite therapeutic advances, metastatic melanoma still accounts for tens of thousands of deaths annually worldwide and represents a small fraction of skin cancer cases but a disproportionate share of cutaneous cancer–related mortality due to its marked aggressiveness and metastatic potential.^2^ Its pathogenesis involves a succession of genetic and epigenetic alterations driven by DNA damage, frequently induced by ultraviolet radiation, which culminate in mutations in critical genes and extensive tumor heterogeneity.^3,4^ From benign lesions, genomic instability and a high mutation rate promote the emergence of cellular subclones with distinct genetic and phenotypic profiles, whose selection during progression to invasive stages is accompanied by increased invasive capacity and intense extracellular matrix (ECM) remodeling.^5^

The interaction between tumor cells and the tumor microenvironment (TME) is a central determinant of melanoma progression.^6^ This complex ecosystem imposes selective pressures, such as hypoxia and acidosis, that favor subclones best adapted to local stress, while simultaneously supporting the establishment of premetastatic niches that prepare distant organs for colonization.^7–9^ Melanoma cells actively remodel the TME and distant tissues through the secretion of soluble factors and vesicles that modulate immune responses, angiogenesis, and ECM dynamics, thereby promoting metastatic dissemination.^8,9^ Metastasis is a highly dynamic process and the tropism of certain tumor types to specific organs dictates the fate of metastatic cells. The dispersion of primary tumors to distant sites relies on a complex network of biological events, ranging from the interaction of adhesion molecules in host cells with their cognate receptors in transformed cells to the molecular reshaping of signaling circuits in the TME.^10–14^ In the case of melanoma, accumulated evidence showed that lymph nodes, lung, liver and brain are common sites for the development of metastasis.^15–17^ Within this context, proteolytic signaling, is a fundamental mechanism in ECM remodeling and premetastatic niche formation.^18^ Proteases irreversibly cleave protein substrates and regulate key processes such as invasion, angiogenesis, metastasis, and immune evasion, whereas imbalances between proteases and their inhibitors favor tumor progression and escape from apoptosis.^19–22^ Degradomic strategies coupled to MS enable the identification of semi tryptic peptides—fragments bearing one terminus generated by *in vitro* tryptic digestion and the other by *in vivo* endogenous proteolysis—whose neo-N- or neo-C-terminal extremities report on cleavage sites and thereby might inform on protease specificity and activity in the TME.^23–25^ The integration of proteomics and degradomics with other omics layers has transformed the understanding of cancer biology and holds promise for precision oncology and personalized therapies^25–27^

To investigate pulmonary melanoma metastasis, the intravenous injection of B16F10 cells into C57BL/6J mice is a well-established syngeneic and immunocompetent model.^28^ This system recapitulates complex tumor–immune interactions, while the B16F10 lineage is characterized by high and reproducible pulmonary metastatic potential, and caudal vein injection closely mimics hematogenous dissemination to the lungs.^28,29^ Compared with xenograft or spontaneous primary tumor models, this approach offers practical advantages for dissecting the biology of metastasis, premetastatic niche formation, and tumor adaptation to the secondary organ.^29^

The dynamic interplay between proteases and their substrates provides a critical framework in oncology, offering substantial potential for the discovery of novel biomarkers and therapeutic targets.Therefore, building on this rationale, the present study provides a degradomic landscape of proteolytic events occurring in pulmonary metastatic lesions, their systemic repercussions in plasma, and the active secretome of tumor cells derived from primary-cultured cells, offering novel insights into the protease-driven mechanisms exploited by melanoma to sustain its progression.

## Material and Methods

### Cell Culture

B16F10 murine melanoma cells (American Type Culture Collection, ATCC, CRL-6475) were cultured in RPMI 1640 medium (Gibco) supplemented with 10% fetal bovine serum (FBS), 2 g/L sodium bicarbonate, 100 units/L penicillin, 100 units/L streptomycin, and 0,25 μg/L amphotericin B. Cultures were maintained at 37 °C in a humidified incubator with 5% CO_2_ and 90% relative humidity. The medium was replaced every 2–3 days, and cells were passaged by enzymatic dissociation with trypsin-EDTA solution when required.

### Injection of Tumoral Cells and Protein Extraction

Eleven-week-old female C57BL/6J mice were obtained from the Center for the Development of Experimental Models for Medicine and Biology (CEDEME, Federal University of São Paulo, UNIFESP, Brazil). Given the high dimensionality and inherent complexity of multicompartment degradomic profiling, focusing on a single sex was essential to ensure a statistically powered characterization of the proteolytic signatures associated with metastasis. This approach minimized confounding biological noise, allowing for a high-resolution mapping of the tumor microenvironment remodeling.

Animals were housed in the animal facility of the Institute of Science and Technology (UNIFESP, São José dos Campos) under controlled conditions (12 h light/dark cycle, 21 ± 1 °C, *ad libitum* access to food and water, three animals per cage) and allowed to acclimate for 5 days prior to experimentation. All procedures were approved by the UNIFESP Committee on Animal Use (CEUA; protocol no. 7974150923) Mice were randomly divided into two groups (n = 6 per group): a control group receiving vehicle and a tumor-bearing group. After intramuscular anesthesia with ketamine (80–100 mg/kg) and xylazine (5–10 mg/kg), the control animals received 100 μL of sterile RPMI medium via tail vein injection, whereas tumor-bearing mice received 1 × 10^6^ B16F10 cells suspended in 100 μL RPMI medium. Animals were monitored twice weekly for clinical signs of distress, including body weight loss and dehydration of skin, coat, or eyes. After 21 days, mice were euthanized via deep sedation with ketamine and xylazine (ketamine 200 mg/kg; xylazine 20 mg/kg) followed by exsanguination. A laparotomy was performed, the abdominal aorta was exposed, the most distal region was punctured, and the thoracic region was massaged to increase blood volume in the vessel. Blood was collected into tubes containing a final concentration of 1% EDTA, and lungs were excised and divided into three portions: (i) snap-frozen in liquid nitrogen; (ii) fixed in 4% paraformaldehyde; and (iii) submerged in RPMI medium with 10% FBS, as described above. Plasma was obtained after blood centrifugation (3000 ×*g* for 15 min at 4 °C) - the upper phase (platelet-poor plasma; ∼250 μL per animal) was collected and stored at −80 °C for further analyses. For protein extraction, frozen lung fragments were homogenized in lysis buffer (0.2 M NaHCO_3_, 0.5 M NaCl, pH 8.3) containing protease inhibitor (SigmaFast, SIGMA) and 1 mM sodium orthovanadate. Lysates were centrifuged at 14,000*g* for 10 min at 4 °C, and supernatants were filtered through 0.22 μm membranes. All samples were stored at −80 °C until use.

### Primary Culture (Tissue Explant) and Secretome Harvesting

Primary culture of cells derived from lung metastatic foci were established after the outgrowth of cells from the tumoral tissue. Briefly, multi well plates (6-wells) were coated with 2 mL FBS for 30 min and, after the removal of coating solution, 1 cm lung tissue fragments were placed (6–8 per well). Each fragment received 50 μL of complete RPMI medium (10% FBS, 2 g/L sodium bicarbonate, antibiotics/antifungal agents as above), followed by an additional 50 μL after 4 h. After overnight incubation, each well received 2 mL of the culture medium. The culture media was replaced every 2–3 days until the cell outgrowth surrounding tissue fragments stabilized. Subconfluent cultures were detached with trypsin-EDTA solution to a T75 culture flask (first passage). At 80% confluence, cultures were washed five times with sterile PBS, and the medium was replaced with serum-free RPMI. After 24 h, the conditioned medium (secretome) was collected and supplemented with protease inhibitors. Samples were centrifuged at 2,200*g* for 10 min at 4 °C, filtered through 0.22 μm membranes, and concentrated using Amicon Ultra-15 centrifugal filters (3 kDa cutoff; Millipore, USA) at 8,000*g*, 4 °C.

### Histological Analysis

Lung fragments were fixed in 4% paraformaldehyde (v/v) at 4 °C for ∼10 days, then washed in running water for 12 h before dehydration. Samples were subjected to graded ethanol series: 30% (30 min), 50% (2 × 30 min), 70% (30 min and 1 h), 95% (2 × 30 min), and 100% (4 × 30 min). Clarification was performed in three successive xylene baths (15, 15, and 30 min, respectively), followed by Paraplast (Sigma, USA) embedding at 55 °C for 1 h, repeated twice to ensure complete infiltration. Embedded tissue was stored at room temperature. Sections (5 μm thick) were obtained using a manual microtome, mounted on Poly-l-Lysine–coated slides (Sigma, USA), and stained with hematoxylin and eosin (H&E) following standard histological procedures.

### In-Solution Trypsin Digestion

Protein samples (200 μg) were subjected to in-solution trypsin digestion as described elsewhere.^13^ Briefly, samples were denatured in 3 M guanidine hydrochloride (GuHCl), reduced with 5 mM dithiothreitol (DTT) for 1 h at 65 °C, and alkylated with 15 mM iodoacetamide (IAA) for 30 min at room temperature in the dark. Excess IAA was quenched with 15 mM DTT for 20 min. Proteins were precipitated by adding 8 volumes of cold acetone (−20 °C) and 1 volume of methanol (−20 °C), incubated at −80 °C for 2 h, and centrifuged at 14,000*g* for 10 min at 4 °C. The pellet was washed twice with cold methanol, air-dried for 5 min and resuspended in 10 mM NaOH, 50 mM HEPES (pH 7.5). Trypsin (Proteomics grade, Sigma, USA) was added at a 1:100 (w/w) enzyme-to-substrate ratio, and digestion proceeded overnight at 37 °C. The reaction was terminated by adding formic acid to a final concentration of 5%. Peptides were desalted using Sep-Pak C18 cartridges (Waters, USA), according to manufacturer’s recommendations. Desalted peptides were dried under vacuum (SpeedVac) and reconstituted in 0.1% formic acid for quantification by BCA assay (ThermoFisher, USA).

### Mass Spectrometry Analysis (LC–MS/MS)

LC–MS/MS analyses were performed on a Vanquish Neo (Thermo Scientific, Waltham, MA, USA) UPLC system coupled to an Orbitrap Exploris 480 mass spectrometer (Thermo Fisher Scientific, Waltham, MA, USA). A total of 300 ng of peptides from each sample was injected onto a PepMap Neo precolumn (Thermo Scientific) containing 5 mm of 5 μm C18 beads, and chromatographic separation of the peptides was performed on a PepMap Neo analytical column (Thermo Scientific) with 150 mm of 2 μm C18 beads, using a 90 min 0.1% formic acid (solvent A)/acetonitrile (solvent B) gradient (0–20% B in 15 min, 20–30% in 60 min,30–40% in 7 min, 40–99%B in 8 min) at a flow rate of 300 nL/min. The mass spectrometer was set to operate at 2.1 kV in positive mode, with the transfer tube temperature set to 270 °C. It was operated in data-dependent acquisition (DDA) mode using high-energy collision-induced dissociation, with a resolution of 60,000 for MS1 precursors, a normalized AGC target of 300%, maximum injection time set to auto, and 30,000 for MS2 fragments. The scan range was set to a 400–1600 *m*/*z* window and positive charge states of 2–6.

### Proteomics Data Processing and Bioinformatic Analyses

Raw LC–MS/MS data were analyzed using FragPipe software^30^ (version 23.1), with MS/MS spectra searched against the *Rodentia* database to account for potential sequence variants and polymorphisms that may not be fully represented in a single-species database, particularly in the context of proteolytic processing and semispecific peptide identification (downloaded from UniProt/SwissProt (accessed on September 09, 2024, containing 38,185 entries). The search parameters included semispecific trypsin specificity, allowing nontryptic cleavage at either the N- or C-terminus, with up to two missed cleavages, carbamidomethylation of cysteines as a fixed modification, and oxidation of methionine and N-terminal acetylation of proteins as variable modifications. The tolerance for precursor and fragment ion masses was set at 20 ppm. The protein and peptide FDRs were set at 1%, with a minimum peptide length of seven amino acids. The variability among biological replicates was assessed using the Pearson correlation coefficient. All bioinformatic analyses were carried out in R environment^31^ (v4.3.1) using in-house scripts. Intensities were log_2_-transformed and missing values were imputed using the left-censored minimal probability (MinProb) method implemented in the *imputeLCMD* package (q = 0.01), which models undetected signals near the lower limit of quantification. Replicate samples were aggregated by computing median intensities for grouped conditions. Pearson correlation coefficients were calculated across all conditions in the complete data set to assess reproducibility. Heatmaps were generated with the *pheatmap* package using row-wise scaling. PCA and discriminant-feature analysis were performed on amino-acid frequency data at the P1 and P1′ positions. Features with zero variance were removed and the remaining matrix was z-score standardized for PCA using *prcomp* in R. Principal component scores (PC1, PC2) and variable loadings were extracted; the top loading vectors (by loading magnitude) were plotted as arrows on a biplot. Separately, group-discriminant features were identified by computing, for each feature and group, the mean frequency inside the group and the mean frequency outside the group, and ranking features by the absolute difference. Positional analysis and inference of active proteases in the samples was carried out using the TopFINDer platform.^32^ To resolve biological pathways associated with proteolytic remodeling, protein substrates from each biological source were subjected to GO Biological Process enrichment analysis. UniProt identifiers were mapped to mouse Entrez IDs using *org.Mm.eg.db* package, and enrichment was computed with *clusterProfiler* package (BH-adjusted *p* < 0.05; *q* < 0.20). The most significant processes were ranked and visualized as dot plots summarizing gene ratio and enrichment strength.

## Results and Discussion

### Metastatic Melanoma Proteome Landscape

Lung tropism after the injection of B16F10 melanoma cells is a well-documented phenomenon^15^ and, as expected, all the animals that received i.v. injection of tumoral cells developed lung metastasis (Figure 1A,B). The widespread melanocytes along lung tissue were evidenced by accumulation of cells at terminal bronchioles and a marked distortion of the tissue architecture (Figure 1C). After the excision of metastatic foci, primary cells were grown—a mixture of tumoral melanocytes and stromal cells (Figure 1D).

**Figure 1:**
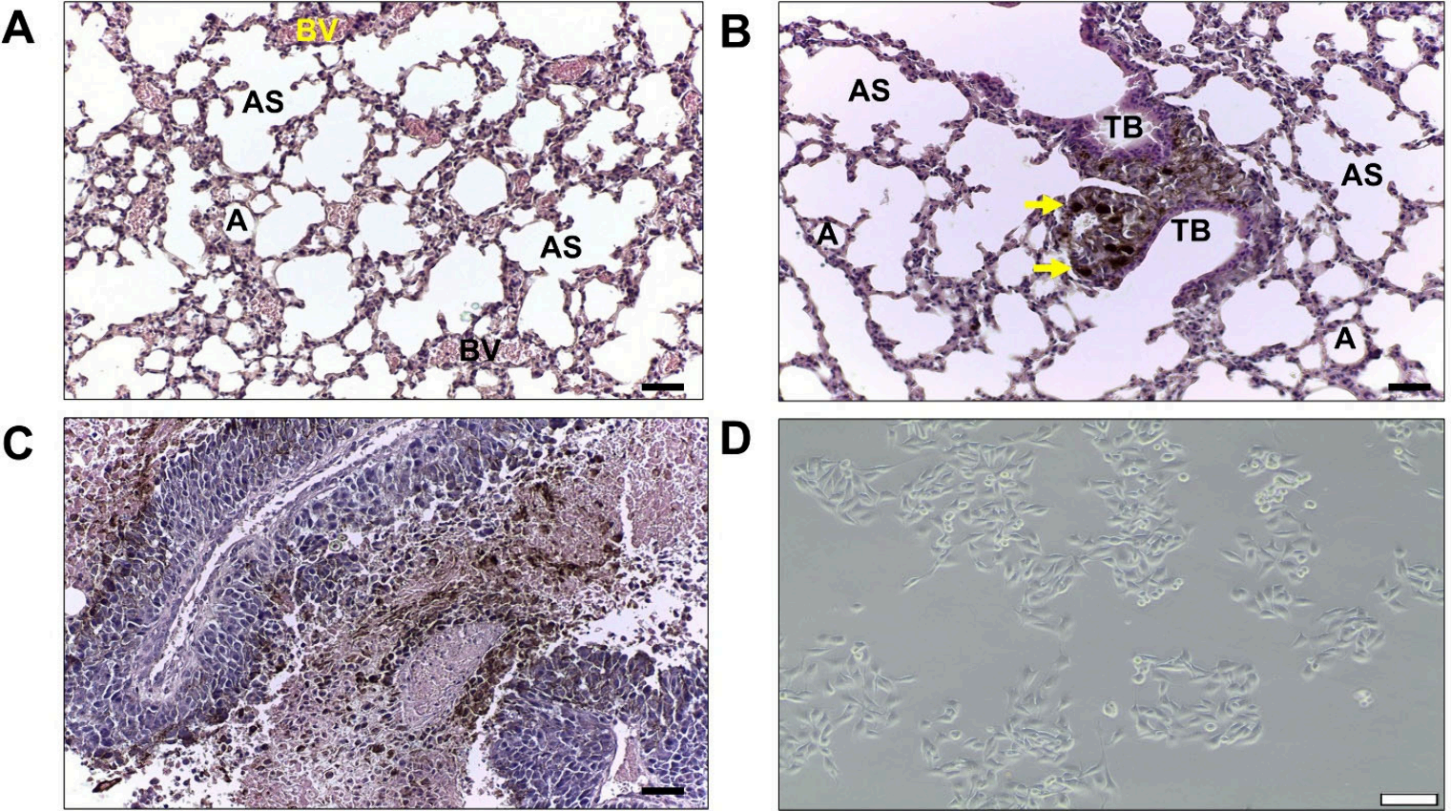
Lung tissue morphology in normal and tumor states. (A) Representative image of normal lung tissue. (B) Representative image of lung tumoral tissue showing melanocyte deposition in the terminal bronchiole (yellow arrows). (C) Altered lung architecture following metastatic colonization. (D) Phase contrast microscope representative image of primary cell culture obtained from lung metastatic foci (all scale bars = 50 μm). (A–alveolus; AS–alveolar sac; B–blood vessel; TB–terminal bronchiole).

Biological samples used in this study were derived from (i) normal and tumoral lung tissue and (ii) plasma; the latter was pooled due to the limited volume obtained from individual animals. The secretome of tumor-derived primary cultures was used as a proxy for the complex signaling occurring at the site of lung metastasis and can be regarded as a snapshot of the tumoral microenvironment during metastatic development.

Primary cell cultures, even when early passaged, may not fully recapitulate the in vivo tumor microenvironment and can introduce culture-dependent effects on cellular behavior and protease expression. Accordingly, tumor-derived primary cultures were used as a controlled system to examine tumor-associated secretory and proteolytic activity rather than to model the full complexity of the metastatic niche. As primary cultures derived from metastatic tissue are inherently heterogeneous, different cell types may contribute to the observed secretory profile; therefore, the present study focused on the integrated secretome of the metastatic microenvironment rather than on cell-type–specific attribution.

Overall, proteomic analysis resulted in the identification of over 2,600 proteins (Supplementary Table 1), with a good correlation among biological samples from each group (Supplementary Figure S1). The highest identification number and protein overlapping were observed in lung tissue and secretome samples (Figure 2A and B).

**Figure 2:**
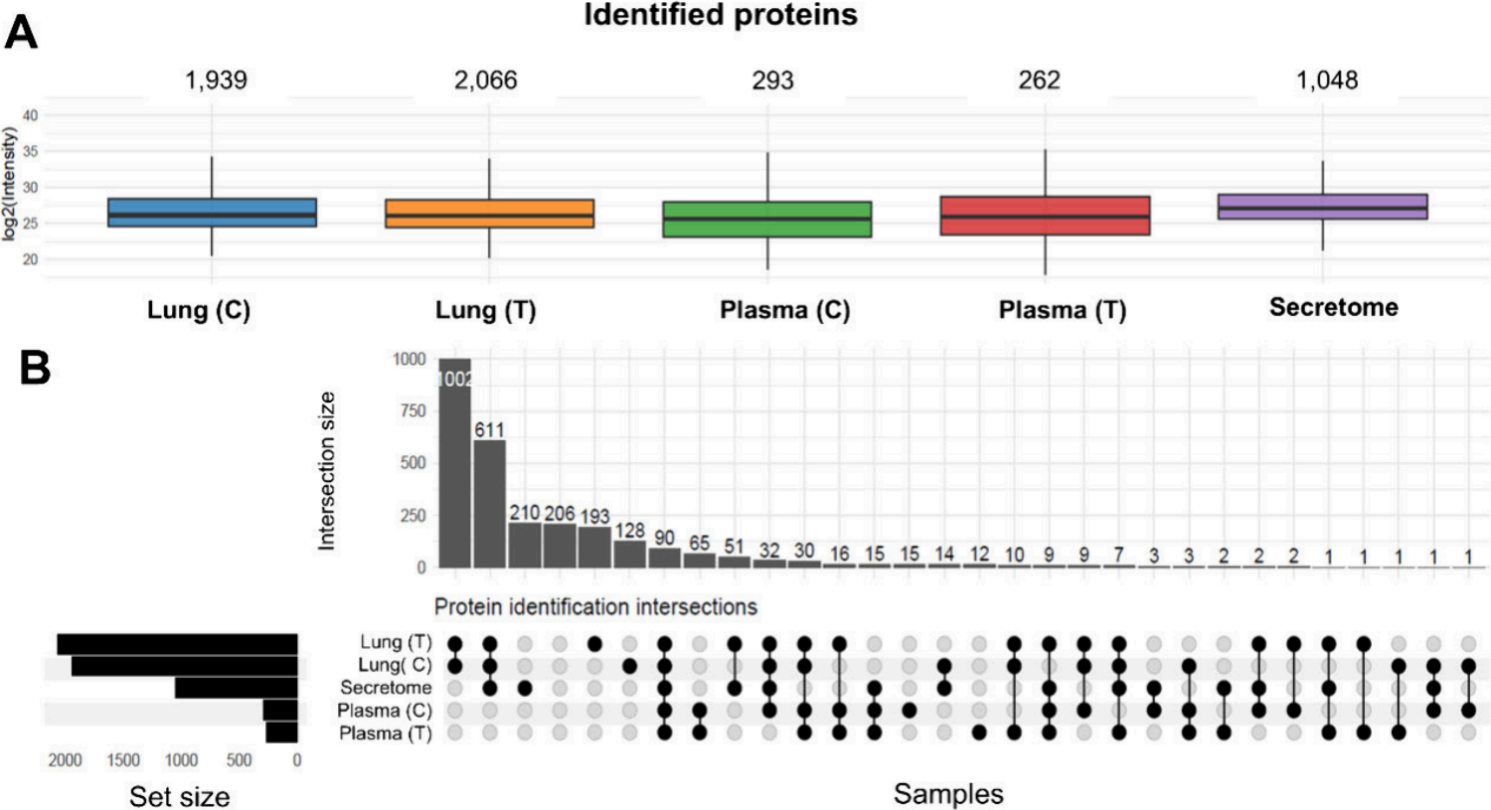
Proteomic coverage and overlap. (A) Protein identifications obtained from the samples analyzed. (B) Overlap of identified proteins across different biological compartments.

Since proteases display a significant role in not only shaping the TME but favoring the spreading of tumoral cells across neighboring tissues,^34,35^ we assessed the abundance profile of all identified proteases and inhibitors in our data set (Figure 3A), including metalloproteases previously implicated in melanoma development and progression.^34^

**Figure 3:**
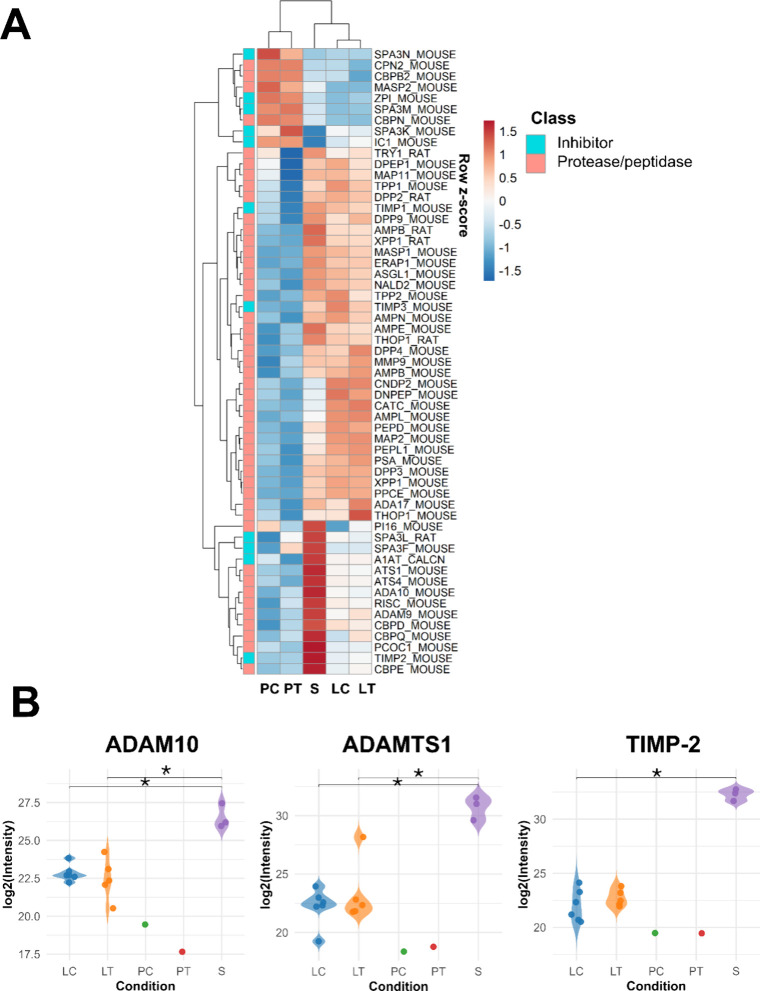
Proteases and protease inhibitors abundance profiles (A) Abundance profiles of proteases/peptidases and their inhibitors in the analyzed biological samples. Protein intensities were log2-transformed and scaled (row z-score). (B) Selected proteases/inhibitors and their corresponding abundance patterns are shown. Replicate-level log2-transformed are shown for each condition. Global differences among LC, LT, and S were evaluated by the Kruskal–Wallis test, followed by Dunn’s posthoc test with Benjamini–Hochberg correction for pairwise comparisons, indicated by brackets and asterisks. PC and PT are shown for descriptive purposes only. (LC = Lung control, *n* = 6; LT = Lung tumor, *n* = 5; PC = Plasma control (pool, 6 animals); PT = Plasma tumor (pool, 5 animals); S = Secretome, *n* = 3;* *p* ≤ 0.05).

Remarkably, the secretome of primary cells, derived from tumoral tissue culture, showed high abundance of metalloproteases involved in the degradation of extracellular matrix components, such as A Disintegrin and Metalloproteinase domain-containing-10 (ADAM10) and A Disintegrin and Metalloproteinase with Thrombospondin motifs 1 (ADAMTS1), as well as the Tissue Inhibitor of Metalloproteinases −2 (TIMP-2; Figure 3B). In this context, the conceptual framework of our proteomics analysis focused on the identification of proteolytic events (i.e., cleavage sites) identified within each biological sample. Our analytical strategy was based on the identification of semi tryptic peptides—those peptides that arose from *in vivo* proteolytic processing. Thus, N- and C-terminal semi tryptic peptides (i.e., peptides lacking trypsin-specific cleavage at either terminus) were considered in database searching parameters of mass spectrometry data. While 44% of the protein identifications relied exclusively on fully tryptic peptides, the remaining 56% were supported by mixed evidence (both fully tryptic and semi tryptic peptides; Supplementary Table 2). This distribution underscores the value of accounting for in vivo cleavage events, particularly in samples where elevated protease activity is anticipated. The contribution of this mixed peptide pool is shown in Figure 4A,B.

**Figure 4:**
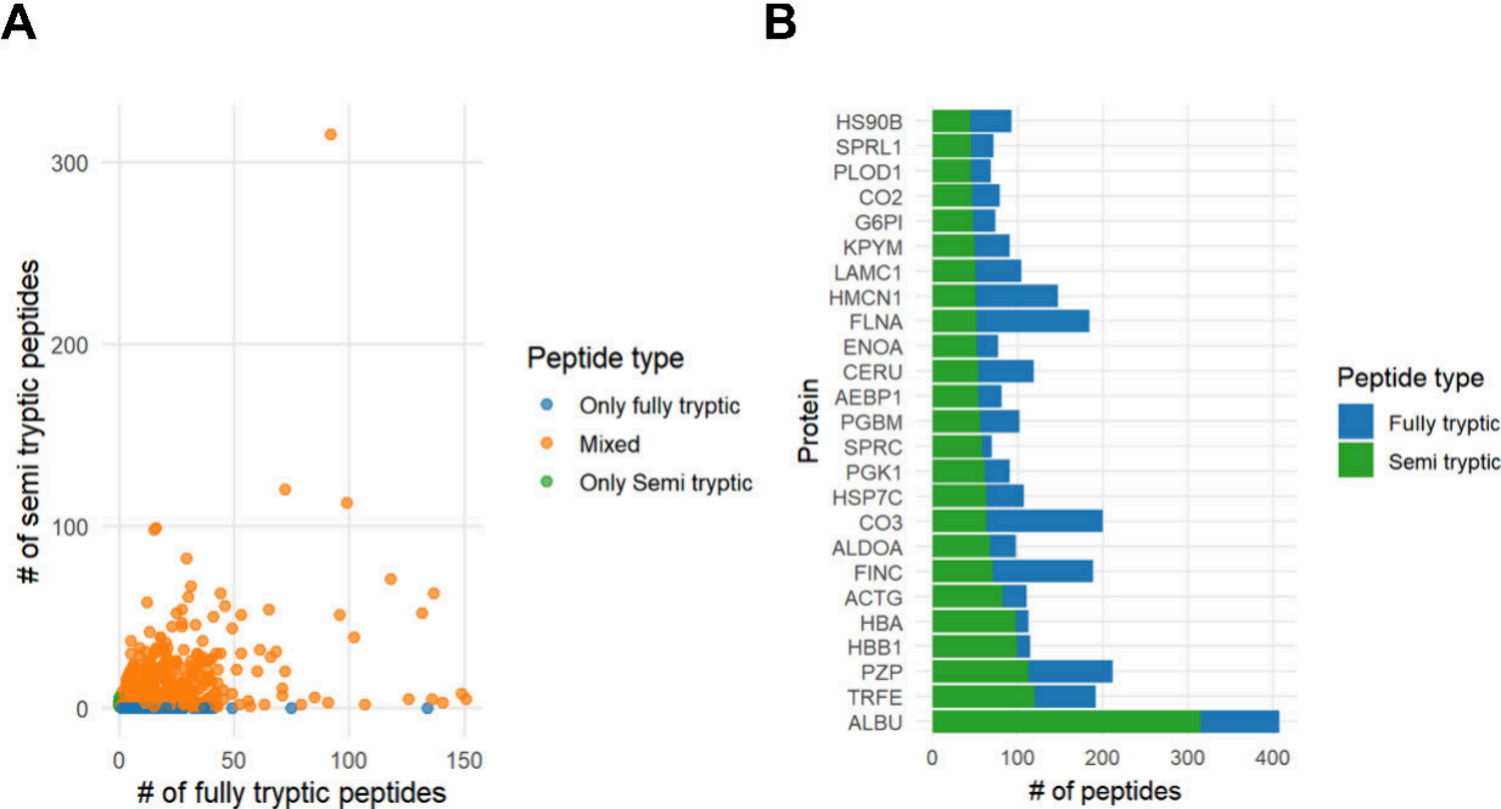
Contribution of distinct peptide types to protein identification. (A) Relative contribution of fully tryptic and semispecific peptides to overall protein identification. Each dot represents a protein and is colored according to the peptide type contributing to its identification. (B) Contribution of mixed peptide types (tryptic and semitryptic) to the identification of selected proteins (top 25 most abundant proteins).

As expected, mixed peptides also contributed to the identification of highly abundant proteins, most notably albumin and hemoglobin (Figure 4B).

### Proteolysis-centric Analysis

Out of the 32,275 peptides identified, 7,936 (approximately 25%) were classified as semitryptic (Supplementary Table 2, Figure 5A). Notably, the secretome derived from the primary cell culture accounted for the largest fraction of semitryptic identifications (49%), followed by lung tumor tissue and tumoral plasma (Figure 5B). Consistent with this pattern, the primary cell culture secretome also exhibited the highest number of unique cleavage sites, suggesting increased proteolytic activity and a broader diversity of protease-specific processing events in this sample type (Figure 5C).

**Figure 5:**
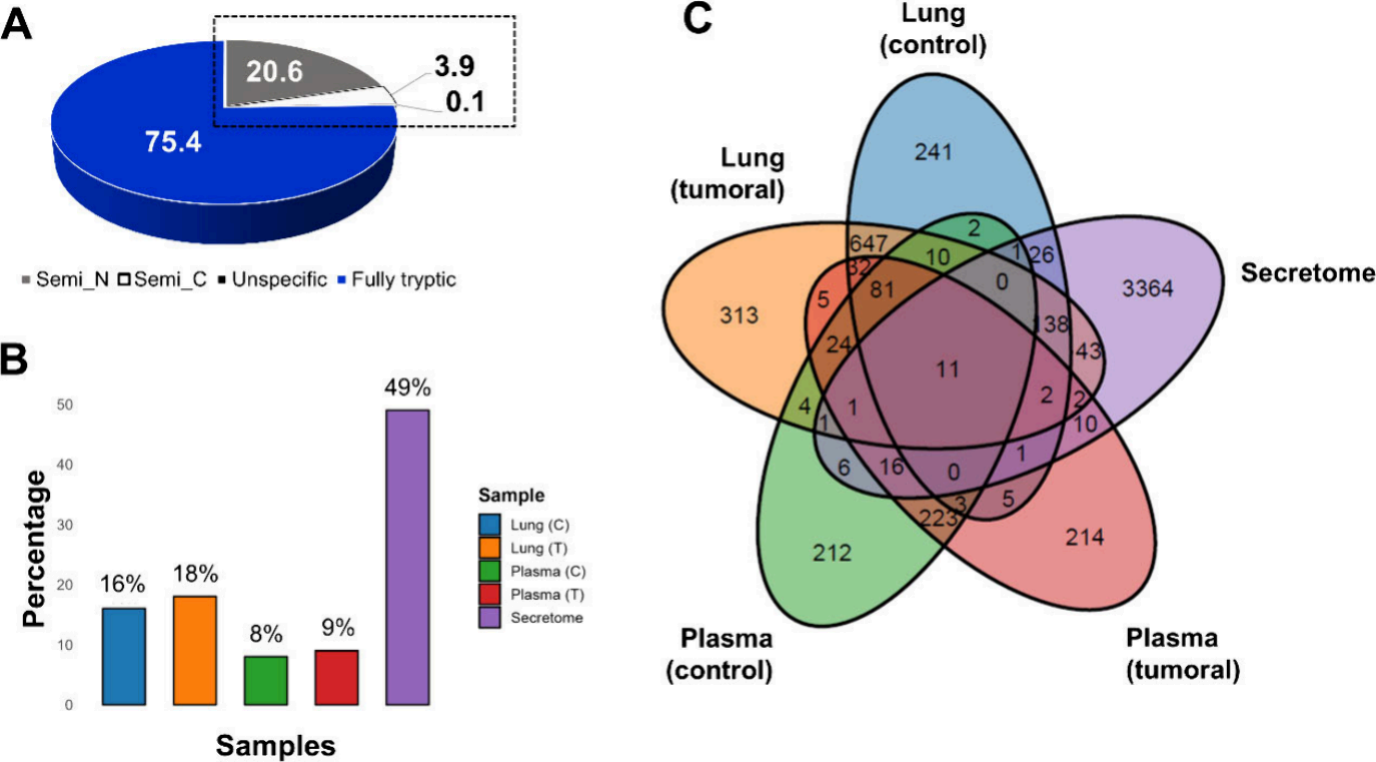
Proteolysis-centric analysis. (A) Proportion of each peptide type to the overall peptide identification (the dashed line corresponds to the semi tryptic/unspecific peptides; Semi_N: Semitryptic peptides at the N-terminus; Semi_C: semitryptic peptides at the C-terminus) and (B) the percentage of semi specific peptides in each biological sample. (C) Venn diagram showing the overlapping of semitryptic peptides across each biological sample.

After removing the highly abundant proteins (albumin and hemoglobin), analysis of the top three most cleaved proteins revealed distinct biological patterns. Actin emerged as the most extensively cleaved protein in metastatic foci (tumoral lung tissue), whereas pregnancy zone protein (α2-macrogobulin) and Secreted protein acidic and rich in cysteine (SPARC) were the primary targets in tumoral plasma and secretome samples, respectively (Figure 6A). Notably, although semitryptic peptides provided broad coverage of all three proteins across both normal and tumoral samples, several peptides were detected exclusively in the tumoral samples (Figure 6B). To further explore actin abundance in lung tissue lysates, we inferred its apparent abundance from fully tryptic peptides derived from control and tumor samples, while excluding semitryptic and nonspecific peptides to avoid bias introduced by proteolytic cleavage–derived fragments. This analysis revealed a lower apparent abundance of actin in tumor tissues, likely reflecting extensive proteolytic cleavage rather than reduced expression (Supplementary Figure S2).

**Figure 6:**
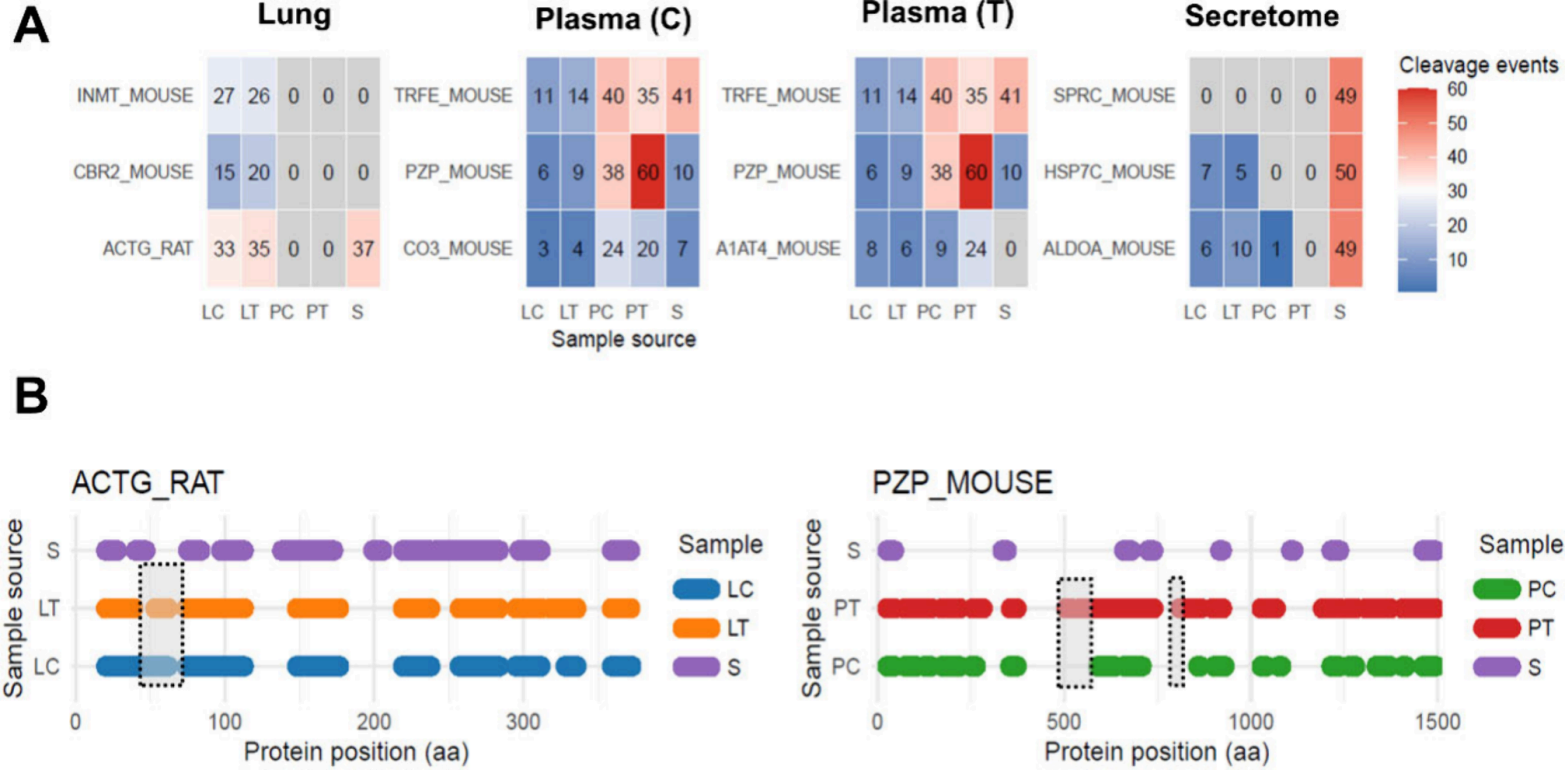
Most cleaved proteins. (A) Representative examples of the three most extensively cleaved proteins identified in each biological sample, excluding albumin and hemoglobin. (B) Graphical representation of cleavage sites in two selected proteins, actin (ACTG_RAT) and α2-macroglobulin (PZP_MOUSE). ACTG_RAT and ACTG_MOUSE share 100% sequence identity; therefore, peptide assignments reflect database annotation rather than species-specific origin. Shaded areas indicate cleavage events detected exclusively in tumor samples.

Although actin is classically regarded as an intracellular protein, accumulating evidence indicates that matrix metalloproteinases, including MMP-2 and MMP-9, can display noncanonical, “moonlighting” functions, as they may localize intracellularly or associate with intracellular compartments under specific physiological and pathological conditions, and several intracellular substrates have already been described.^36–39^ Actin cleavage may promote cytoskeletal remodeling, facilitating tumor cell motility and invasion within the lung tumor tissue.^40^ α2-macroglobulin is a protease inhibitor that acts as broad spectrum- protease “traps” and can carry cytokines, growth factors, and other ligands.^41^ Cleavage of α2-macroglobulin might reflect both consumption/inactivation of protective α--macroglobulins by tumor driven proteases, representing systemic proteolytic changes supporting tumor progression. Moreover, its cleavage could promote the release of trapped biological molecules into the metastatic niche, therefore, contributing to the proteolytic shaping of metastasis. SPARC is a matricellular protein that modulates cell-matrix interaction;^42^ thus, its cleavage in the secretome of primary cultured cells may reflect an extracellular matrix–remodeling program that remains active even in early passage cultures derived from metastatic sites—a process fundamental to invasion and metastatic niche formation. In addition, cryptic functions may appear after its cleavage; cleaved SPARC fragments have been implicated in modulating cell adhesion, migration, and invasion, impacting metastatic potential.^43–45^ Taken together, these cleavage patterns likely capture ongoing proteolytic activity and structural remodeling across cellular, systemic, and microenvironmental levels, all of which may contribute to lung melanoma metastasis progression and colonization.

Analysis of unique peptides (cleavage sites exclusive to each condition) revealed a conserved pattern across all experimental conditions. Examination of amino acid frequencies at positions P3 to P3′ of the substrates (following the Schechter and Berger nomenclature^46^) showed consistent trends among the sample types. Except for the secretome, all samples exhibited a strong preference for histidine at P1 and serine at P1′ (Figure 7). Small, hydrophobic residues, particularly alanine and leucine, were predominant at both P2 and P2′ positions, respectively. In contrast, the secretome displayed dominant leucine and serine residues at the P1 and P1′ positions, respectively. According to the MEROPS peptidase database,^47^ the cleavage motifs represented by His-Ser and Leu-Ser are typical for some cathepsins, including Cathepsin F, L, K, and others. Cathepsins are present and often upregulated or activated in lung tissue, especially under inflammatory, fibrotic, infectious, or metastatic conditions.^48–50^ Therefore, this cleavage pattern may reflect the expected physiological degradomic profile of lung environment.

**Figure 7:**
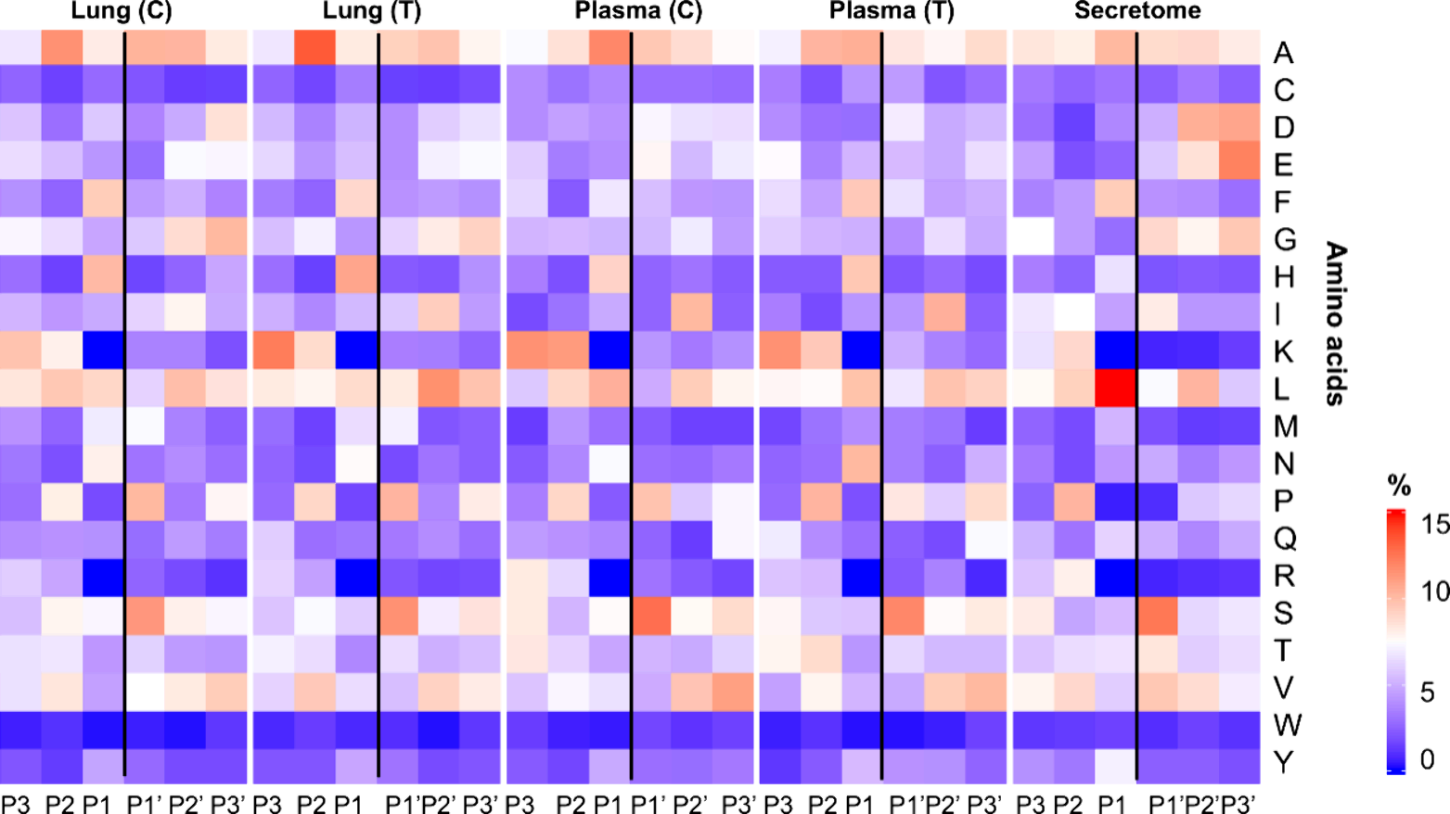
Proteolytic signatures across tissues and compartments. Heatmaps show amino-acid frequencies surrounding cleavage sites (P3–P3′ positions) for each biological condition: Lung (C), Lung (T), Plasma (C), Plasma (T), and Secretome.

To explore this further, we performed a multivariate analysis of the cleavage preferences at the P1 and P1′ positions, aiming to identify potential signatures capable of stratifying the samples not only by their origin (lung, plasma, or secreted proteins) but also by more subtle distinctions that might otherwise be obscured by the dominant pattern (Figure 8A). Discriminant feature analysis identified distinct molecular signatures that differentiate each experimental group (control and tumoral lung samples, control and tumoral plasma, and secretome sample). Based on the absolute difference parameter (“abs_diff”; Supplementary Table 3), which quantifies the magnitude of separation between within-group and out-group means independently of direction, the three most discriminant features were determined for each group and emphasized consistent and group-specific separation patterns (Figure 8B).

**Figure 8:**
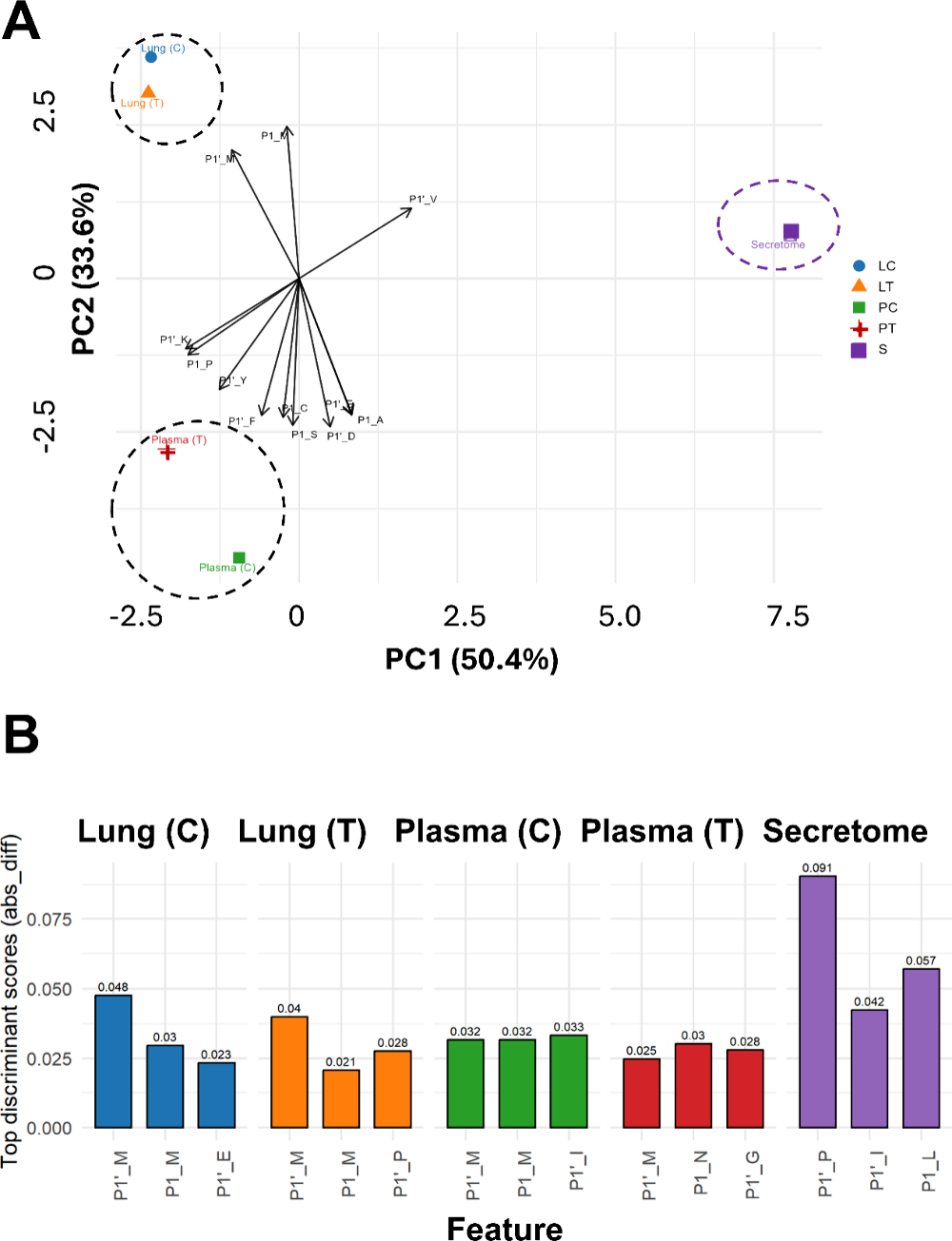
Discriminant-feature analysis uncovered unique proteolytic signatures across sample types. Composite figure combining PCA biplot (A), showing sample clustering and top loadings and bar plots (B) displaying the top three discriminant Position–Amino acid features for each sample group, ranked by the absolute difference between within-group and out-of-group mean frequencies (abs_diff). Each panel corresponds to a single group and its associated discriminant score. These features highlight the most pronounced, group-specific shifts in amino-acid usage at P1 or P1′, revealing residues that best distinguish each condition within the degradomic landscape.

Glutamate and proline at the P1′ position emerged as the main discriminators for control and tumoral tissues, respectively. Control and tumoral plasma samples also displayed distinct cleavage signatures: tumoral plasma was characterized by polar and small residues—asparagine at P1 and glycine at P1′—whereas control plasma predominantly showed isoleucine at the P1′ position. The secretome (S group) exhibited the greatest discriminative magnitude, indicating a particularly pronounced divergence in feature expression compared with other conditions, with leucine at P1 and proline and isoleucine at P1′. Collectively, these results underscore molecular patterns and their ability to capture biologically meaningful variability across the experimental data set.

### Functional Implications of the Cleavage Events in Lung Metastasis and the Prognostic Use of Proteolytic Signaling in Melanoma

Gene Ontology (GO) enrichment analysis of the substrates identified after the exclusive peptide (cleavage sites) mapping revealed distinct functional signatures across biological samples. In metastatic foci (lung tumor tissue), substrates associated with general catabolic processes—particularly glucose catabolism—were significantly enriched (Figure 9A). Cleavage of substrates related to acute inflammatory response and negative regulation of wound healing processes predominated in tumoral plasma (Figure 9B). In contrast, substrates involved in cell–substrate adhesion and matrix remodeling were overrepresented among those cleaved in the tumoral secretome (Figure 9C).

**Figure 9:**
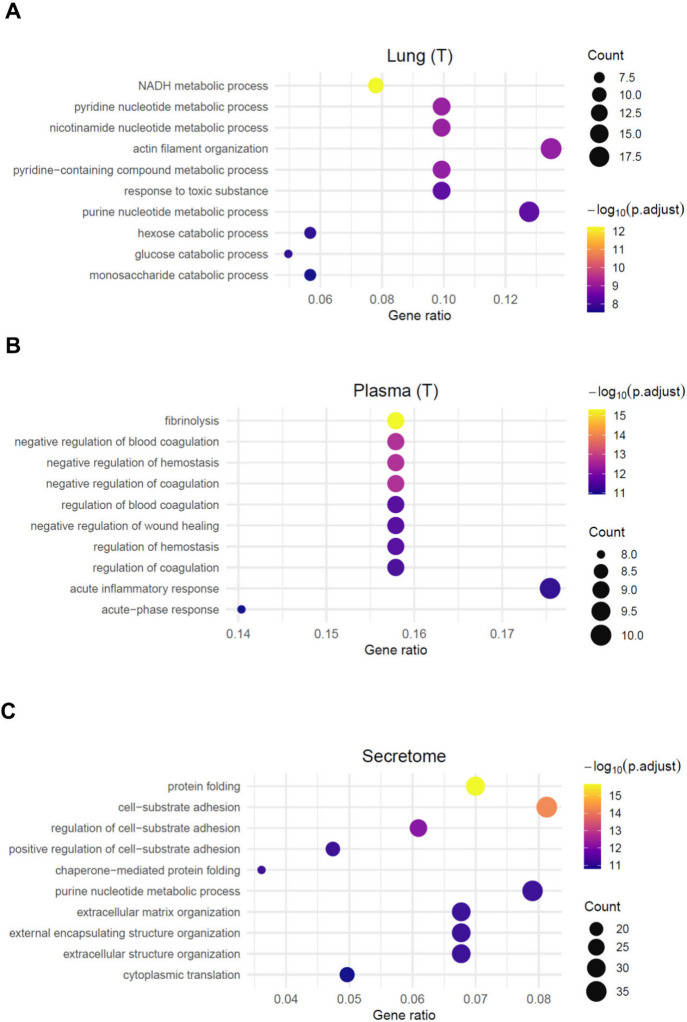
Functional implications of substrates identified by peptides exclusive to tumor samples. Shown is the Gene Ontology (GO) Biological Process enrichment analysis of the substrates identified by peptides from tumoral lung (A), tumoral plasma (B) and secretome (C).

From a functional standpoint, these substrate profiles suggest that proteolytic remodeling during metastasis contributes to the dynamic reorganization of both the tumoral and systemic environments, influencing key metabolic and structural pathways that sustain disease progression. When evaluated in combination with the cleavage specificity (as illustrated in Figure 8), these results indicate differential protease activity across biological compartments. To better characterize the proteases implicated in these cleavage events, we employed this same set of unique peptides to interrogate established protease–substrate specificities via the TopFINDer platform^32^ (Supplementary Table 4). It is important to note, however, that protease inference based on curated databases such as TopFIND is inherently influenced by the depth of annotation available for individual proteases, which may bias predictions toward well-characterized enzymes. Although no annotated cleavage events were detected in the peptide set derived from tumor-associated plasma, the mapped cleavage sites were consistent with the activity of at least 11 proteases whose specificities have been reported in previous studies (Figure 10). Notably, a pronounced proportion of cleavage sites from MMP2, Cathepsin E and Cathepsin D were detected in both tumoral lung tissue and in the secretome of the primary cell culture. Indeed, MMP2 and cathepsins such as CATD have well-documented roles in promoting melanoma metastasis through extracellular matrix (ECM) degradation, epithelial-mesenchymal transition (EMT) induction, and immune modulation,^51–53^ affecting tumor progression and metastasis. Taken together, these observations integrate our findings on P3–P3′ amino acid frequencies, multivariate analyses, and cleavage specificities, and indicate a pronounced reorganization of the proteolytic landscape within the lung metastatic niche in murine melanoma.

**Figure 10:**
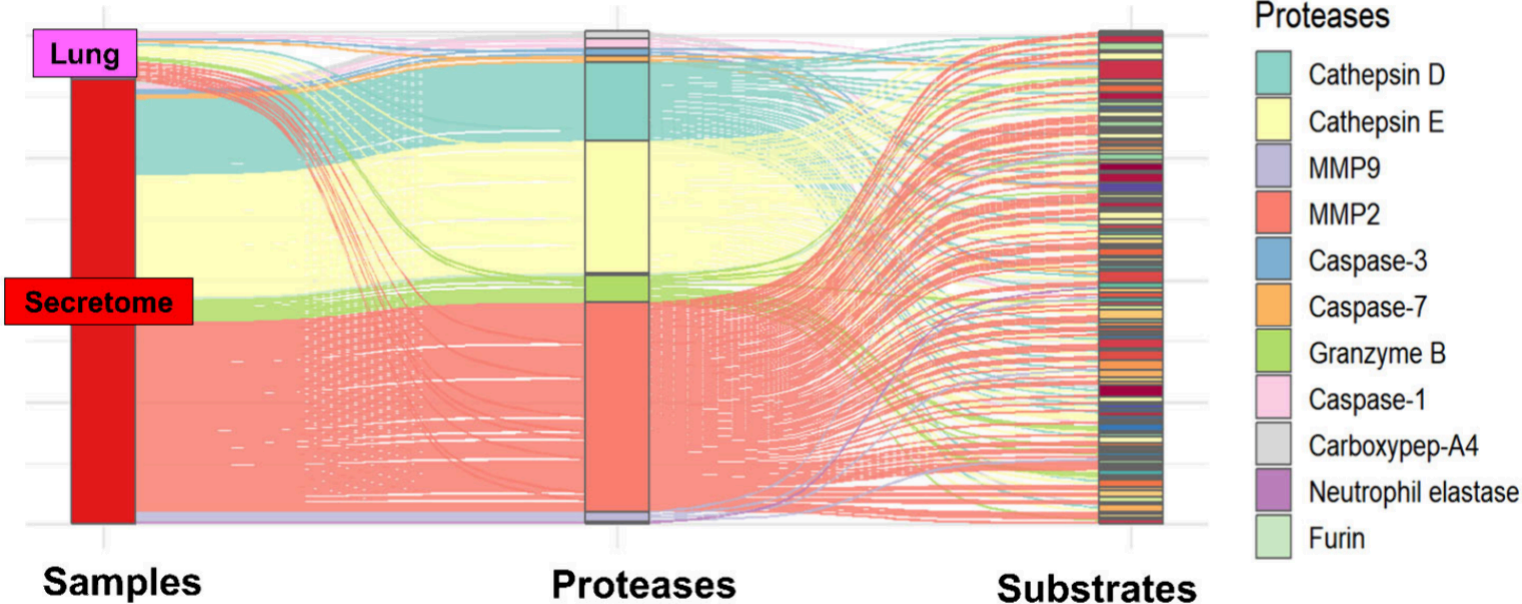
Potentially active proteases in tumoral samples. Alluvial plot showing the proteases whose activity was inferred by mapping of the cleavage sites (exclusive semitryptic peptides) from tumoral samples in TopFINDer platform.^32^

In addition to the degradomic and in silico protease inference analyses, functional evidence supporting the involvement of matrix metalloproteases was obtained by gelatin zymography using SDS–polyacrylamide gels containing gelatin, which revealed gelatinolytic activities consistent with the presence of active gelatinases, compatible with MMP-2 and MMP-9 (Supplementary Figure S3). Moreover, immunoblot analysis further supported the presence of MMP2 at the protein level (Supplementary Figure S4).

## Conclusions

This study provides a discovery-level characterization of proteolytic signatures associated with metastatic melanoma in a murine model by integrating degradomic profiling across lung tissue, plasma, and tumor-derived secretome. Despite the comprehensive nature of our multicompartment degradomic analysis, this study has limitations that should be acknowledged. Our experiments were conducted exclusively using female mice to ensure experimental homogeneity and to maximize the statistical power required for high-dimensional proteomic profiling. While this approach allowed for a robust identification of proteolytic signatures in the tumor microenvironment, it does not account for potential sex-specific differences in melanoma progression and systemic remodeling. Given that sex is a critical biological variable in cancer immunology and metastasis, future studies incorporating male cohorts are warranted to validate these degradomic patterns across a broader biological context.

The use of semispecific database searching enabled a global assessment of proteolytic processing and revealed compartment-specific cleavage patterns associated with melanoma progression. Our results indicate that semitryptic peptides can serve as biochemical footprints of protease activity occurring at distinct stages of disease dissemination, from local tissue remodeling in the lung to systemic alterations detectable in plasma. Notably, the secretome of early passaged tumor–stromal cultures reflect a highly active proteolytic environment that may contribute to the establishment of the metastatic niche. As an exploratory investigation, this work establishes a robust framework for hypothesis-driven studies and provides a valuable degradomic resource. While further biochemical and translational validation will be required to deepen functional interpretation, the patterns described herein support the hypothesis that protein cleavage events are temporally coordinated with melanoma progression and may hold relevance for future prognostic applications.

## Supplementary Material





## Data Availability

The mass spectrometry proteomics data have been deposited to the Mass Spectrometry Interactive Virtual Environment (MassIVE, https://massive.ucsd.edu/ProteoSAFe/static/massive.jsp) with the data set identifier: MSV000100089.

## Notes

Supplementary Table 1: Output of FragPipe search (combined_protein.txt file). Supplementary Table 2: Output of FragPipe search (combined_peptide.txt file). Supplementary Table 3: Discriminant features (top12) per group (based on PCA analysis of P1/P1’ positions for each group). Supplementary Table 4: Peptide mapping results (output from TopFINDer analysis) (XLSX)
Figure S1: Pearson correlation analysis of (log2-transformed) protein intensities across biological replicates. Figure S2: Actin abundance is decreased in lung tumor lysates. Figure S3: Gelatin-zymography of representative samples in 12% SDS-PAGE under nonreducing conditions. Figure S4: Immunoblot analysis (anti-MMP2) (PDF)
