# Supplementary material for: A Degradomic Landscape of Proteolytic Remodeling in Melanoma Lung Metastasis
Source: J Proteome Res. 2026 Feb 25;25(3):1749–60. doi: 10.1021/acs.jproteome.5c01205 (PMC12973369; doi:10.1021/acs.jproteome.5c01205)
Supplement: Supplementary file 2 [file pr5c01205_si_002.pdf]

SUPPORTING INFORMATION

A degradomic landscape of proteolytic remodeling in melanoma lung metastasis

Camila Eduarda Alves Martins Nogueira[1], Fabiana Olímpio[2], Murilo Salardani[1], Uilla Barcick[1], Luis Roberto Fonseca Lima[1], Flávio Aimbire[2,3] Letícia Dias Lima Jedlicka[1,4], Flávio V. Loures[5], Luciane Portas Capelo[6], Valdeci Lima[6], Taysa Monteiro de Oliveira[6] Bianca C.S.C. Barros[7,8], Solange M.T. Serrano[7], André Zelanis[1,*]

[1]Functional Proteomics Laboratory, Federal University of São Paulo – UNIFESP,12231-280, São José dos Campos, São Paulo, Brazil;

[2]Department of Medicine, Postgraduate Program in Translational Medicine, Federal University of São Paulo (UNIFESP), Rua Pedro De Toledo 720 - 2° Andar, Vila Clementino, São Paulo, SP, 04039-002, Brazil.

[3]Department of Science and Technology, Lab. Immunopharmacology, Federal University of São Paulo (UNIFESP), Rua Talim, 330, Vila Nair, São José dos Campos, SP, 12231-280, Brazil.

[4]Instituto de Estudos em Saúde e Biológicas, Universidade Federal do Sul e Sudeste do Pará-Unifesspa,68507-590, Marabá, Pará, Brazil;

[5]Applied Immunology Laboratory, Institute of Science and Technology, Federal University of São Paulo, Rua Talim, 330, Vila Nair, São José dos Campos, SP, 12231-280, Brazil.

[6] Laboratory of Bone Development and Bioengineering, Federal University of São Paulo – UNIFESP,12231-280, São José dos Campos, São Paulo, Brazil;
[7]Laboratory of Applied Toxinology, Center of Toxins, Immune-Response, and Cell Signaling (CeTICS), Butantan Institute, 05503-900, São Paulo, Brazil, São Paulo-SP, Brazil;
[8]Laboratory of Cellular Immunology and Biochemistry of Fungi and Protozoa, Postgraduate Program in Pharmaceutical Sciences, Federal University of Sao Paulo (UNIFESP), 09913-030,Diadema, São Paulo, Brazil.
*Corresponding author:

André Zelanis
andre.zelanis@unifesp.br
Functional Proteomics Laboratory
Department of Science and Technology
Federal University of São Paulo (ICT-UNIFESP)
São José dos Campos, São Paulo, Brazil
Rua Talim, 330, Sala 202, 12231-280;
Tel +55 11 3385-4135 ext. 9727.

Supplementary figures.pdf

- Supplementary figure S1: Pearson correlation analysis of (log2-transformed) protein intensities across biological replicates.
- Supplementary figure S2: Actin abundance is decreased in lung tumor lysates.
- Supplementary figure S3: Gelatin-zymography of representative samples in 12% SDS-PAGE under non-reducing conditions.
- Supplementary figure S4: Immunoblot analysis (anti-MMP2).

Supplementary tables.xls

- Supplementary table 1: Output of FragPipe search (combined_protein.txt file)
- Supplementary table 2: Output of FragPipe search (combined_peptide.txt file)
- Supplementary table 3: Discriminant features (top12) per group (based on PCA analysis of P1/P1' positions for each group).
- Supplementary table 4: Peptide mapping results (output from TopFINDer analysis. For details, see material and methods section of the main text).

.

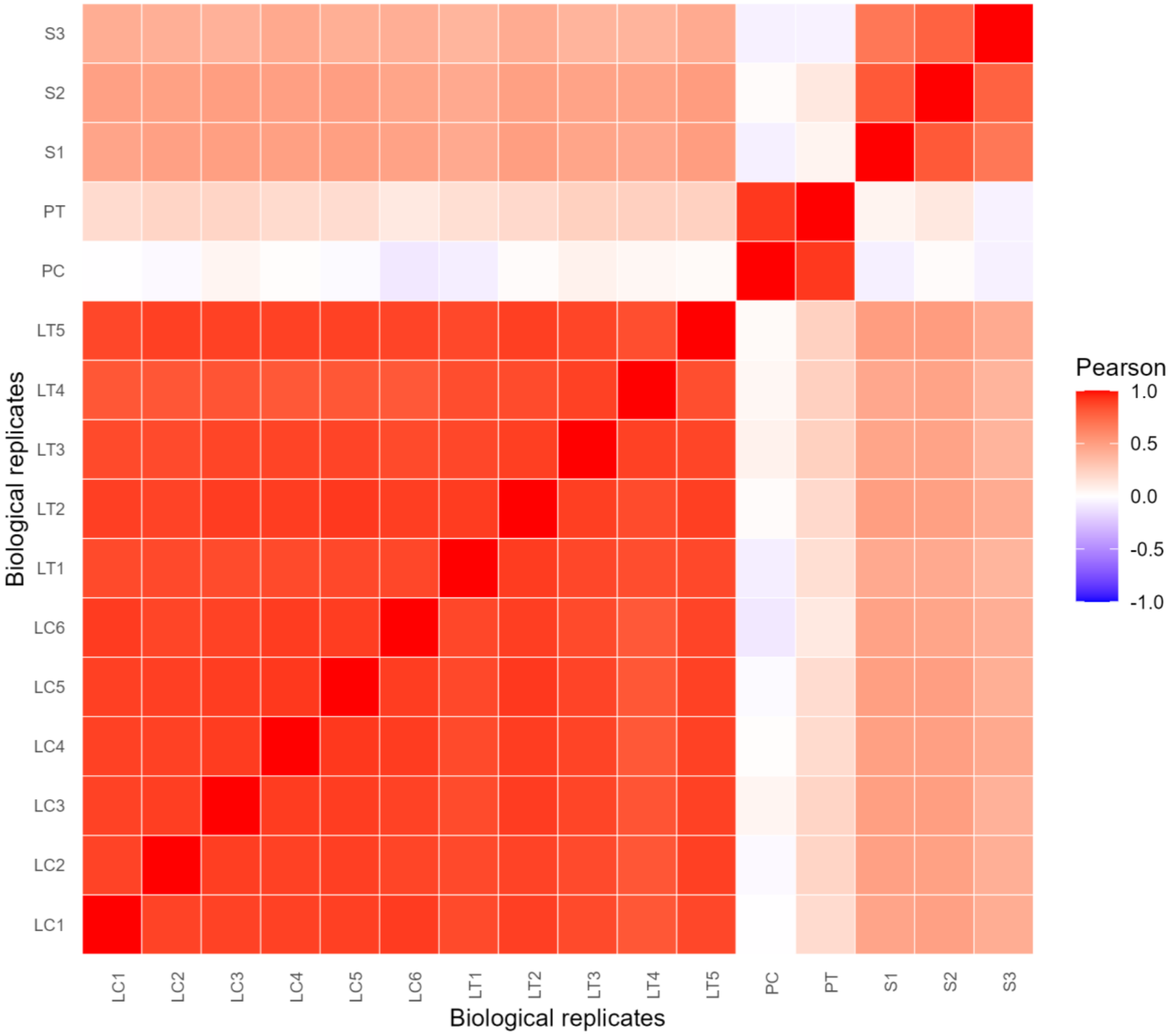

Supplementary figure S1: Pearson correlation analysis of (log2-transformed) protein intensities across biological replicates.

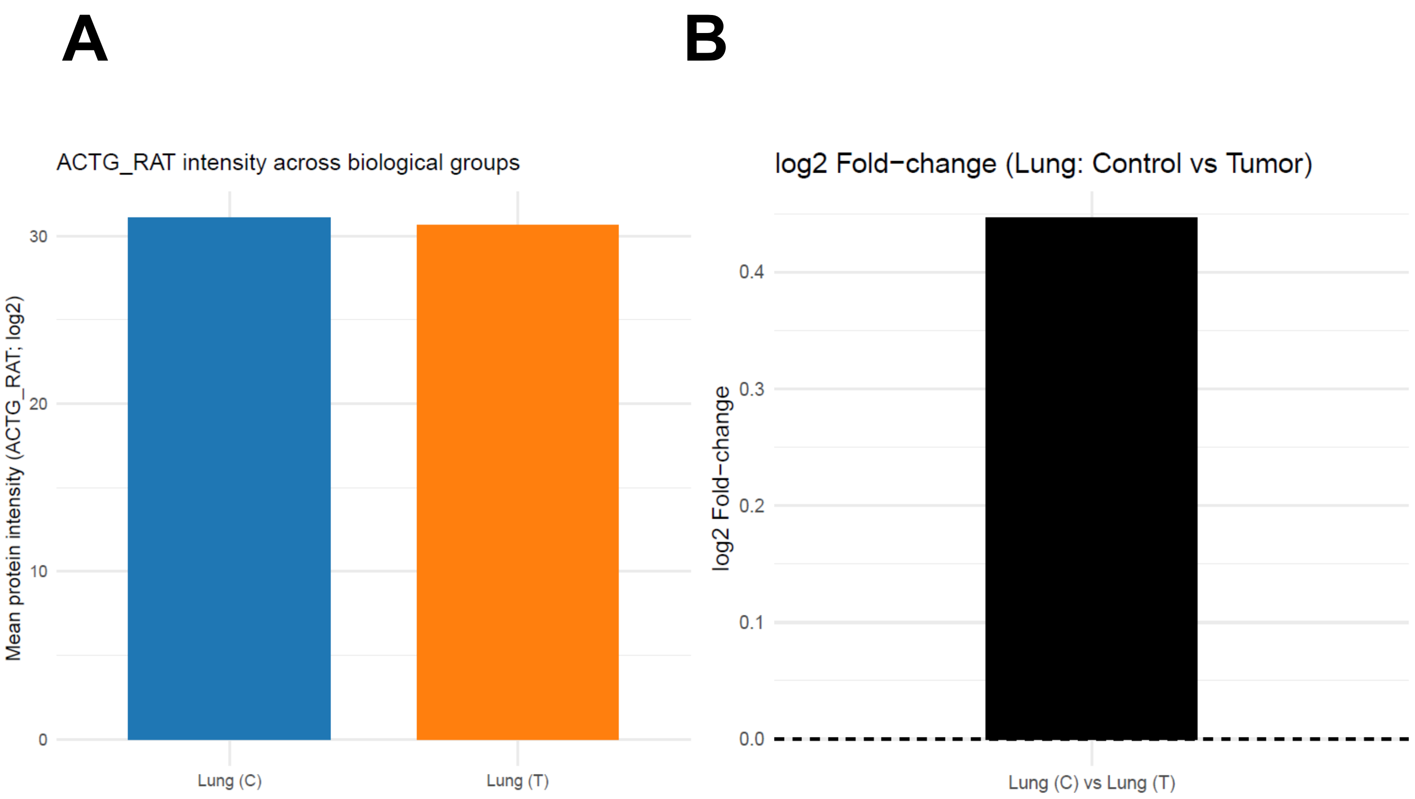

Supplementary Figure S2. Actin abundance is decreased in lung tumor lysates. **A.** Apparent actin abundance in lung tissue lysates was estimated using exclusively fully tryptic peptides derived from control and tumor samples. **B.** Tumor lung tissue displayed a lower apparent actin abundance, consistent with extensive proteolytic processing rather than reduced protein expression, as indicated by the positive log2 fold change (Control/Tumor).

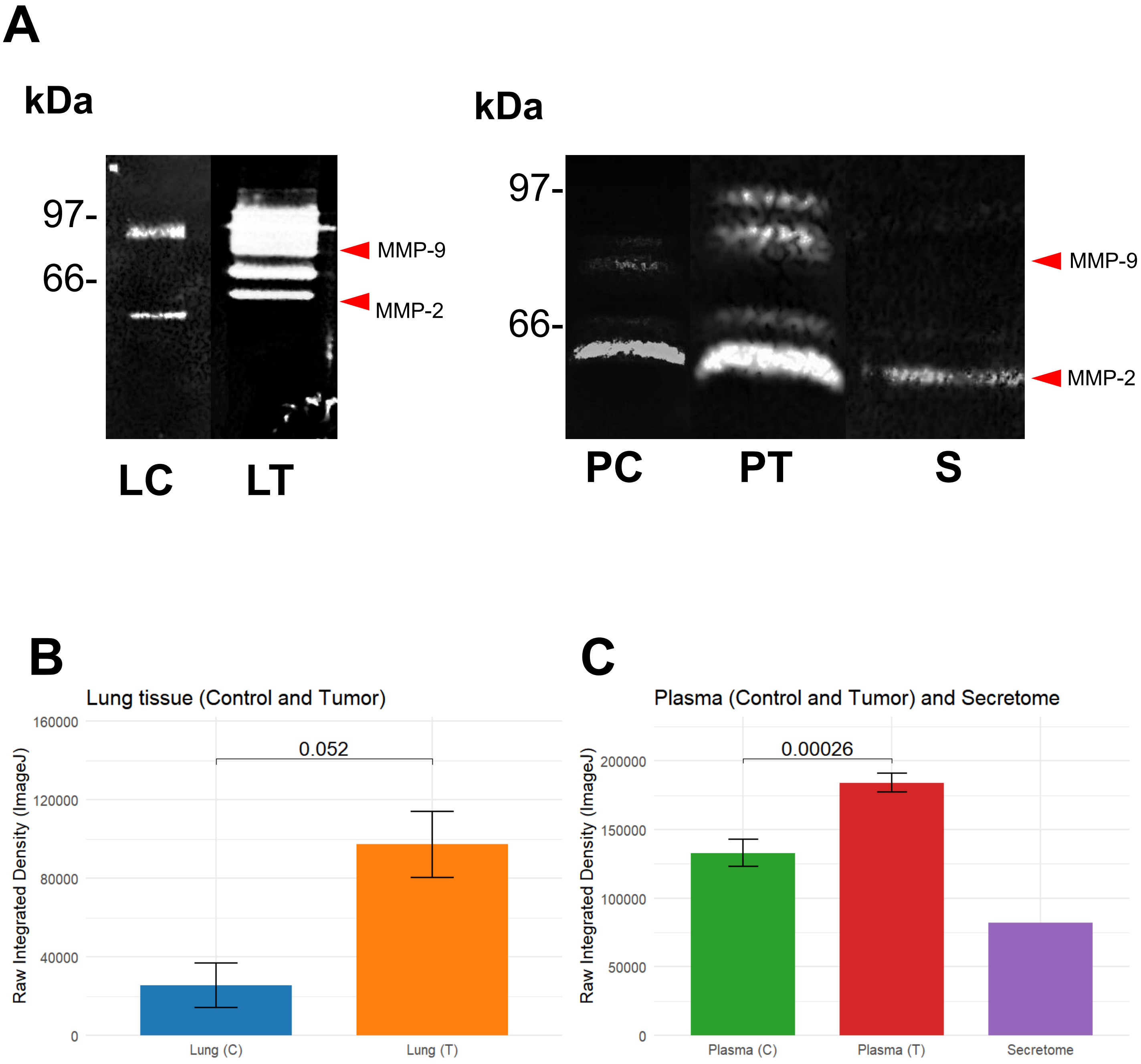

Supplementary Figure S3. (**A**) Gelatin zymography performed on 12% SDS–PAGE under non-reducing conditions. Representative samples are shown: pooled lung tissue lysates from controls (LC; n = 6; 20 µg protein) and tumors (LT; n = 5; 20 µg protein); pooled plasma samples from controls (PC; n = 6; 20 µg protein) and patients with tumors (PT; n = 5; 20 µg protein); and primary culture secretome (S; n = 3; 6.5 µg protein). Red arrows indicate the approximate molecular weight range of active MMPs. (**B** and **C**) Quantitative analysis of the MMP-2 band detected by gelatin zymography in lung tissue, plasma, and secretome samples. Band intensities were quantified by densitometry using ImageJ software and expressed as raw integrated density. Bars represent mean ± SD for each group. Statistical comparisons between control and tumor groups were performed using an unpaired t-test.

A

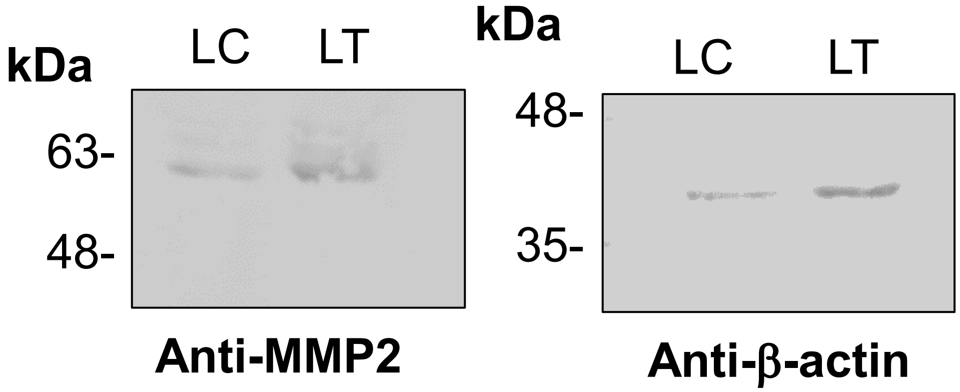

B

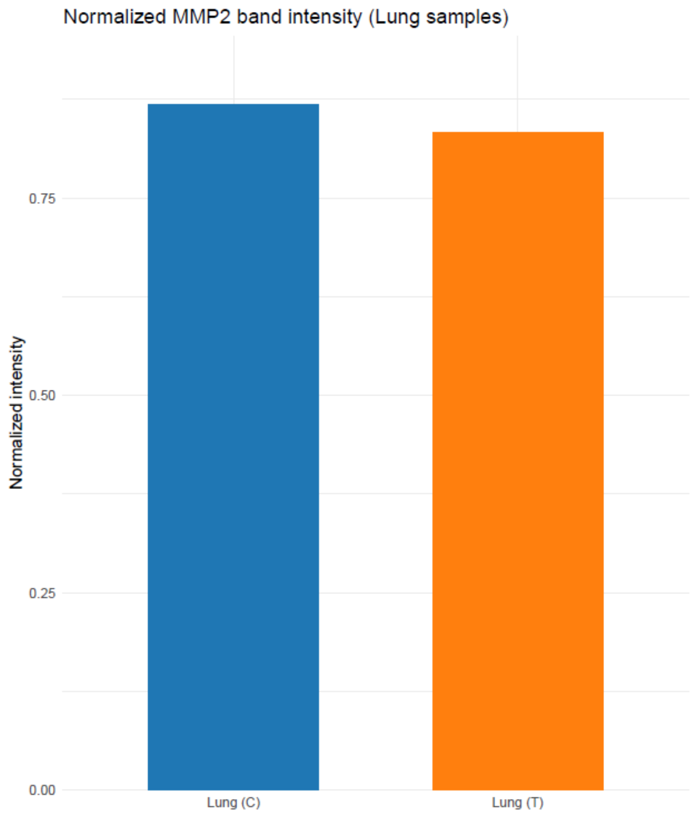

Supplementary figure S4:A. Immunoblot analysis. was performed using proteins separated by 12% SDS–PAGE under reducing conditions and transferred to a nitrocellulose membrane. Representative pooled samples (30 µg per lane) included lung control lysate (LC) and lung tumor lysate (LT). Anti-MMP2 and anti-β-actin primary antibodies (1:250; Abcam, UK) and donkey anti-rabbit IgG–HRP secondary antibody (1:250; Novex, Thermo Fisher Scientific, USA) were used. Immunoreactive bands were visualized using 4-chloro-1-naphthol (4CN), a chromogenic substrate for horseradish peroxidase (HRP). **B**. Quantification of Western blot band intensities. Densitometric values were obtained using ImageJ software, normalized according to the loading control (β-actin) and expressed as relative intensity for lung control and tumor samples. Bars represent normalized values for each biological sample.
